# Development of Functional Kiwifruit Jelly with *chenpi* (FKJ) by 3D Food Printing Technology and Its Anti-Obesity and Antioxidant Potentials

**DOI:** 10.3390/foods11131894

**Published:** 2022-06-26

**Authors:** Mingfang Peng, Zhipeng Gao, Yanfang Liao, Jiajing Guo, Yang Shan

**Affiliations:** 1 Key Laboratory of Agro-Products Processing, Key Laboratory of Agro-Products Quality and Safety Control in Storage and Transport Process, Ministry of Agriculture and Rural Affairs of China, Beijing 100193, China; pmf5246@163.com; 2 International Joint Lab on Fruits & Vegetables Processing, Quality and Safety, Hunan Key Lab of Fruits & Vegetables Storage, Processing, Quality and Safety, Hunan Agriculture Product Processing Institute, Hunan Academy of Agricultural Sciences, Changsha 410125, China; lyfdeng@163.com; 3 Longping Branch, Graduate School of Hunan University, Changsha 410125, China; 4 College of Animal Science and Technology, Hunan Agricultural University, Changsha 410128, China; gaozhipeng627@163.com

**Keywords:** anti-obesity, antioxidant, *chenpi*, functional food, kiwifruit

## Abstract

With the growing popularity of the concept of healthy diet, modern obesity treatment is gradually shifting from surgical or pharmacological treatment to nutritional intervention. As a safe and effective measure, natural product interventions are a potential strategy of obesity management. The present study aimed to develop a kind of functional food rich in bioactive compounds (*chenpi*, kiwifruit, and pectin as raw materials) and investigate their bioactive effects on a mouse model. For development of functional kiwifruit jelly with *chenpi* (FKJ), the results of single-factor and response surface experiments showed that the optimized formulation was composed of a 30.26% addition of *chenpi*, 35% addition of kiwifruit juice, and 2.88% addition of pectin. The FKJ obtained with the optimal formulation could be used as a 3D printing raw material to print the desired food shapes successfully. For bioactivity evaluation of FKJ, the results with a mouse model showed that the food intake, liver weight, and adipose tissue weight were significantly decreased after administration of FKJ with dose-dependent effect compared to the CON group (*p* < 0.05). Meanwhile, the serum levels of several inflammatory factors (TG, IL-6, and TNF-α) were decreased and the activities of several antioxidant-related enzymes (SOD, GSH-PX, and CAT) were increased. In short, a functional kiwifruit jelly with *chenpi* was developed in this study. It is a functional snack food rich in active phenolic compounds, low in calories, with antioxidant and anti-inflammatory activity, and prevents fat accumulation. FKJ could well meet the needs of modern people for nutrition and health and also promote the processing and utilization of natural products, and has good development prospects in the functional food industry.

## 1. Introduction

The increasing incidence of obesity worldwide has become a significant public health problem that threatens the health of people around the world [1]. Obesity increases the risk of developing metabolic syndromes, such as heart disease, hypertension, dyslipidemia, and diabetes [2]. With the change in lifestyles, the treatment of obesity is gradually shifting from drug treatment to dietary intervention and prevention [3]. Studies have shown that obesity is closely related to oxidative stress [4,5] and that dietary supplementation with antioxidants has a potential therapeutic effect on weight loss or control [6,7]. Therefore, there is an urgent need to develop functional foods that offer long-term prevention of obesity and antioxidant activity.

Aged citrus peel, also known as *chenpi*, is usually made from the mature pericarp of *Citrus reticulata*. *Chenpi* contains various physiologically active ingredients, such as volatile oils, flavonoids, alkaloids, and polysaccharides [8]. A growing number of studies have found that *chenpi* can prevent obesity [9], hepatic steatosis, and metabolic syndrome [9,10], improve blood lipid profiles [11], and modulate liver and heart function parameters [12]. Kiwifruit (*Actinidia chinensis Planch*) is a ubiquitous and popular fruit in our daily life. It is rich in vitamin C [13] and other bioactive substances, such as phenolic compounds [14], insoluble fiber, carotenoids, flavonoids, and minerals [15]. Kiwifruit has a highly antioxidant effect and immunostimulatory activity in vitro and in vivo [16]. In addition to being eaten fresh, kiwifruit is often used to produce various functional foods, such as dried fruit snacks [17], fruit bars [18], gold kiwifruit leather products (a snack made of fruit puree and other ingredients) [19], kiwifruit milk powders [20], and kiwifruit jellies [15]. Jellies are considered an excellent substitute for fruit processing, which can greatly meet our requirements for the development of FKJ. Low methoxylated pectin (LMP) could form a gel state with calcium in an "ionotropic gelation" manner [21] and is often used as a raw material for production of jelly. Studies have shown that pectin could prevent and control obesity and diabetes [22], as well as improve lipid and cholesterol metabolism [23]. Pectin from citrus peel is an important by-product of citrus processing, thus its use in the production of jellies may be a good option for citrus waste management.

In the current lifestyle, people tend to prefer foods that are ready to eat and contain enough nutritional ingredients. As shown above, *chenpi*, kiwifruit, and LMP exhibit great potential for the development of functional food; thus, we expected to develop a kind of functional kiwifruit jelly with *chenpi*. Meanwhile, 3D food printing technology was used in the development process to meet the personalized needs of consumers. Further, the bioactivities of FKJ were explored. We expect that FKJ developed in this study will provide a good reference for the development of functional foods and provide a better choice for food intake that meets consumer demand.

## 2. Materials and Methods

### 2.1. Materials

*Chenpi* was purchased from Jiangmen, Guangdong Province, made by red *Pericarpium Citri Reticulatae* dried in natural sunlight and sealed for 3 years. Low methoxylated pectin (LMP) from citrus fruits (purity > 98%) was purchased from Yantai Andre pectin Co., Ltd. (Yantai, China). Kiwifruit (Zespri green kiwi) was purchased from Changsha, China. All were food-grade materials. Total antioxidant capacity kits (ABTS method, DPPH method, FRAP method) were purchased from Suzhou Comin Biotechnology Co., Ltd., Suzhou, China. All kits for the determination of serum biochemical indexes were purchased from Jiangsu Meimian Co., Ltd., Yancheng, China.

### 2.2. Development of FKJ

#### 2.2.1. Processing of FKJ

FKJ was produced by modifying and combining the methods proposed by Nuramalia, D.R. et al. and Lima, M.B et al. [24,25]. In brief, 10 g of crushed *chenpi* was added to 200 mL of purified water and boiled for 40 min, then filtered with vacuum filter while hot. The filtrate was concentrated to 10 mL using a rotary evaporator to obtain 1 g/mL of *chenpi* decoction. The kiwifruit juice was obtained by squeezing and filtering after peeling the kiwifruit. Citrus pectin was added to 10 mL of purified water while stirring, while the pectin was dissolved in a water bath at 70 °C for 10–15 min, after which different volumes of *chenpi* decoction and kiwifruit juice were added, stirred well, and 1 mL of 0.14 g/mL CaCl_2_ solution was added. Then, purified water was added to reach a total volume of 100 mL [26]. Finally, the solutions were mixed evenly, subjected to pasteurization at 75 °C for 5 min, cooled, shaped, and stored at 4 °C.

#### 2.2.2. Sensory Evaluation

The sensory analysis of FKJ was conducted by a sensory evaluation team, which consisted of ten food professionals. The total sensory evaluation score consisted of four components: color, tissue state, flavor, and taste. The sensory scoring criteria are shown in Table 1.

#### 2.2.3. Single-Factor Experiments (SFE)

Single-factor experiments were designed to investigate the effects of different additions of *chenpi* decoction, kiwifruit juice, and pectin on the sensory evaluation score of FKJ. They could provide a range of factor levels for the design of response surface methodology. In our study, the effect of calcium was not analyzed, since its primary role was to cross-link pectin to form a gel state, and 1 mL of 0.14 g/mL CaCl_2_ solution was added to all jelly formulations [27]. *Chenpi* decoction content, which was the main functional ingredient, was selected as 10–35%. Kiwifruit juice content, which could increase the flavor of FKJ and mask some of the bitterness of the *chenpi*, was chosen as 15–40%. The pectin content, which determines the gel state of FKJ and increases the toughness while maintaining the jelly shape, was chosen as 1–6% as referenced and adapted from Khouryieh, H.A, et al. [28]. Samples were prepared by adding different levels of *chenpi* decoction, kiwifruit juice, and pectin according to Section 2.2.1. The total score was given by the sensory evaluation team after sensory identification.

#### 2.2.4. Experimental Design of Response Surface Methodology (RSM)

The response surface methodology can optimize the process by evaluating the factors affecting biological processes and their interactions [29]. The range of each factor in the response surface was selected from the results of the single-factor experiments. Three factors and three levels were used according to the central combination experimental design principle of the Box–Behnken model. *Chenpi* decoction, kiwifruit juice, and pectin addition were selected as the independent variables A, B, and C, respectively, and represented by 1, 0, and −1 for each of the three levels. The total score of the sensory evaluation was used as the response value (R) to determine the best formula for FKJ based on regression analysis. This production recipe was used in subsequent experiments. The specific parameters of RSM are shown in Table 2.

#### 2.2.5. 3D Food Printing of FKJ

The aim of this assay was to obtain designed FKJ shapes through 3D printing and investigate whether the food formulations developed in our study could be personalized through 3D food printing. The best food recipe obtained by the response surface method was used to prepare samples, which were then used as food inks for printing. The food inks were sent to a specialized 3D food printing company (Hangzhou Shiyin Technology Co., Ltd.) for 3D printing tests. The 3D printing process is based on the extrusion of food inks at room temperature [26]. A 3D printer with a nozzle diameter of 0.84 mm was used for printing tests (Foodbot S2, Hangzhou Shiyin Technology Co., Ltd., Hangzhou, China). The exact shape of the print was designed by Cura15.02.1 software before printing (cuboid: 2.5 × 2.5 × 1.25 cm^3^). The parameters of the printing process on the printer were set (infill velocity V: 15 mm/s, infill density: 90%, infill pattern: rectilinear, print temperature: 40 °C) according to Vancauwenberghe V. et al. [26].

### 2.3. Determination of Nutrients and Bioactive Components of FKJ

The ash (using GB 5009.4-2016 method) [30], moisture (using GB 5009.3-2016 method) [31], protein (using GB 5009.5-2016 method) [32], fat (using GB 5009.6-2016 method) [33], carbohydrate, and sodium content (using GB 5009.91-2017 method) [34] of FKJ were determined to obtain the main nutritional composition.

The total flavonoids in FKJ were determined by the aluminum nitrate chromogenic method, which was adapted from Li X. et al. [35]. A 0.3 mL amount of FKJ solution, 4.7 mL of 30% ethanol, and 0.4 mL of 5% NaNO_2_ solution were mixed well and reacted for 6 min. After that, 0.4 mL of 10% Al(NO)_3_ solution was added and mixed well, standing for 6 min, then 4 mL of 4% NaOH solution was added and supplemented with 30% ethanol to make a total volume of 10 mL and mixed well. After 3 min, the absorbance values were measured by a UV-Vis spectrophotometer at 510 nm, and the total flavonoid content was presented as mg/g rutin equivalent (RE), using a rutin equivalent (0–0.24 mg/mL) standard curve.

The total phenolic concentrations were assayed using the Folin–Ciocalteu method modified from Yamin et al. [36]. A 10 µL amount of FKJ solution, 4.90 mL of distilled water, and 0.5 mL of Folin–Ciocalteu reagent were combined in a colorimetric tube and then shaken well. The mixture was placed in the dark for 5 min. After that, 2.5 mL of 5% Na_2_CO_3_ solution was added, then distilled water was added to make a total volume of 10 mL. The total solution was mixed well, then left for 60 min in the dark. The absorbance was measured at 765 nm by a UV-Vis spectrophotometer, and the total phenolic content was expressed as mg/g of gallic acid equivalent (GAE). Gallic acid solution in the range of 0–0.08 mg/mL was used to determine the standard curve.

### 2.4. Measurement of Antioxidant Capacity of FKJ

The total antioxidant capacity of FKJ was determined by three frequently used assays, including 2,2-diphenyl-1-octanohydrazide (DPPH) and 2,2-azinobis (3-ethyl-benzothiazoline-6-sulfonic acid) (ABTS) radical scavenging activity, and ferric reducing antioxidant power (FRAP) [37]. The results were presented as µmol/g of Trolox equivalents (TE). In this experiment, FKJ used for the ABTS scavenging assay was diluted 80 times with extraction buffer from the kit, while FKJ used for the DPPH scavenging assay and FRAP reduction test was diluted 50 times. Finally, these assays were performed following the kit instructions.

### 2.5. Determination of Bioactivities of FKJ Using Mouse Model

#### 2.5.1. Animal Administration and Treatment

Forty healthy male C57BL/6J mice (seven weeks old) were purchased from Hunan SJA laboratory animal Co., Ltd. (Changsha, China). They were housed in a controlled environment with a light/dark cycle of 12 h, temperature of 21–24 °C and relative humidity of 50–80%, with food and drinking water available ad libitum. The animal experiment was carried out after the review and approval of the Hunan Agricultural University Institutional Animal Care and Use Committee (202005).

After one week of acclimatization, all mice were randomly allocated into four groups (*n* = 10): Control (CON), High-dose (KH), Medium-dose (KM), and low-dose (KL). The mice in the KH, KM, and KL groups were administered 10 g/kg, 5 g/kg, and 2.5 g/kg FKJ daily by gavage, respectively. The CON group was gavaged with an equal volume of saline simultaneously. All groups were provided with a standard diet (composed of 54.9% corn, 18% soybean meal, 5.6% casein, 6.5% beer yeast, 0.7% lard, 0.8% bean oil, 0.5% salt, 1.4% fishmeal, and 1% premixture) during the experimentation. Body weight and food intake of each mouse were recorded weekly throughout the experiment. At the end of the experiment in week 8, the mouse blood was collected from the orbital sinus. Liver, subcutaneous adipose tissues (SAT), abdominal adipose tissues (AAT), and perirenal adipose tissues (PEAT) were collected and weighed.

#### 2.5.2. Histopathological Observation

Paraformaldehyde-fixed AAT samples were dehydrated with ethanol, embedded with paraffin, and sectioned for hematoxylin and eosin (H&E) staining [38]. The pathological sections were observed and photographed using a light microscope (Nikon Eclipse E100, Tokyo, Japan) at 40× magnification.

#### 2.5.3. Biochemical Analysis of Serum

Blood was allowed to stand for 4 h at 4 °C and then centrifuged at 3000 rpm for 15 min using a freezing centrifuge, and the supernatant was carefully separated and stored at −80 °C for testing [39]. The levels of total cholesterol (TC), triglycerides (TG), low-density lipoprotein cholesterol (LDL-C), and high-density lipoprotein cholesterol (HDL-C) in the serum were analyzed. The indicators of liver function were assessed, including alanine aminotransferase (ALT) and aspartate transaminase (AST). The serum concentrations of interleukin-6 (IL-6) and tumor necrosis factor-alpha (TNF-α) were assessed, and markers of antioxidant capacity were measured, including malonaldehyde (MDA), superoxide dismutase (SOD), glutathione peroxidase (GSH-PX), and catalase (CAT). All of these biochemical indicators were determined according to the instructions of corresponding kits purchased from Jiangsu Meimian Co., Ltd. (Yancheng, China).

### 2.6. Statistical Analysis

Design-Expert (version 13) software was used for the design of response surface experiments. All of the data were plotted by Origin Pro 9.0 software. The IBM SPSS Statistics 26.0 software was used for comparative analysis. Student’s *t*-test was used to compare the data between the two groups, and the two-way analysis of variance (ANOVA) was used for the comparative analysis of multiple groups of data. *p* < 0.05 was indicated as a significant difference.

## 3. Results

### 3.1. Development of FKJ

#### 3.1.1. Single-Factor Experiment

**Figure 1:**
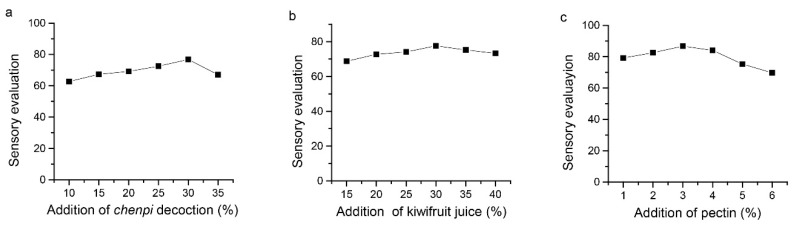
Effects of *chenpi* decoction (**a**), kiwifruit juice (**b**), and pectin (**c**) on sensory evaluation of FKJ.

As shown in Figure 1a, increasing the addition of *chenpi* decoction resulted in increasing the sensory evaluation score of FKJ (Figure 1a). This growth trend continued up to 30%, whereas with higher additions of *chenpi* decoction, the sensory evaluation score declined. When we carried out the single-factor experiment with kiwifruit juice as the independent variable based on the addition of 30% *chenpi* decoction, the same trend as that of *chenpi* decoction was observed (Figure 1b). The results showed that the best addition of kiwifruit juice was 30%. Based on this result, we kept the additions of 30% *chenpi* decoction and 30% kiwifruit juice unchanged to investigate the effect of pectin concentration on sensory evaluation of FKJ. The sensory evaluation of FKJ showed an increasing trend with increased additions of pectin from 1 to 3%, whereas further increases in added pectin resulted in a decrease in the sensory evaluation score (Figure 1c). Therefore, the response surface design level of the *chenpi* decoction was 25–35%, the level of kiwifruit juice was 25–35%, and the level of pectin was 2–4%.

#### 3.1.2. Response Surface Analysis

The development of FKJ was optimized using the response surface methodology (Box–Behnken design). The experimental design and related results are shown in Table 3. Using the total sensory evaluation score as the response variable (R), the regression analysis between R and three independent variables yielded the following regression equation:

R = −611.63 + 40.48 A − 1.85 B + 57.88 C + 0.01 AB − 0.1 AC − 0.05 BC − 0.67 A^2^ + 0.06 B^2^ −9.25 C^2^ (1)

It was possible to predict the response values under different independent variables using the model equation. Table 4 shows that the model had a *p*-value < 0.0001, and the lack of fit had a *p*-value > 0.05, suggesting that the values predicted with the established regression model were extremely significant. In this model, the predicted R² of 0.9090 was in rational consistence with the adjusted R² of 0.9784, indicating that the actual sensory evaluation values fitted well with the predicted values. This model had a coefficient of variance (CV) of 2.49% (<10%) and adequate precision value of 23.96 (>4), which showed excellent reproducibility and accuracy.

ANOVA analysis revealed that factor B (kiwifruit juice) showed an extremely significant difference (*p* < 0.0001) and factor C (pectin) was significantly different (*p* < 0.05), but factor A (*chenpi* decoction) was not significant (*p* = 0.0679). Larger F-values indicated a more significant effect of the factor on the response value. Therefore, the effect of each factor on the sensory evaluation of the product could be ranked as B-kiwifruit juice > C-pectin > A-*chenpi* decoction. Analysis of the *p*-values of the factors in the model showed that A^2^ and C^2^ had extremely significant differences (*p* < 0.0001), while the remaining effects were not significant (*p* > 0.1).

**Figure 2:**
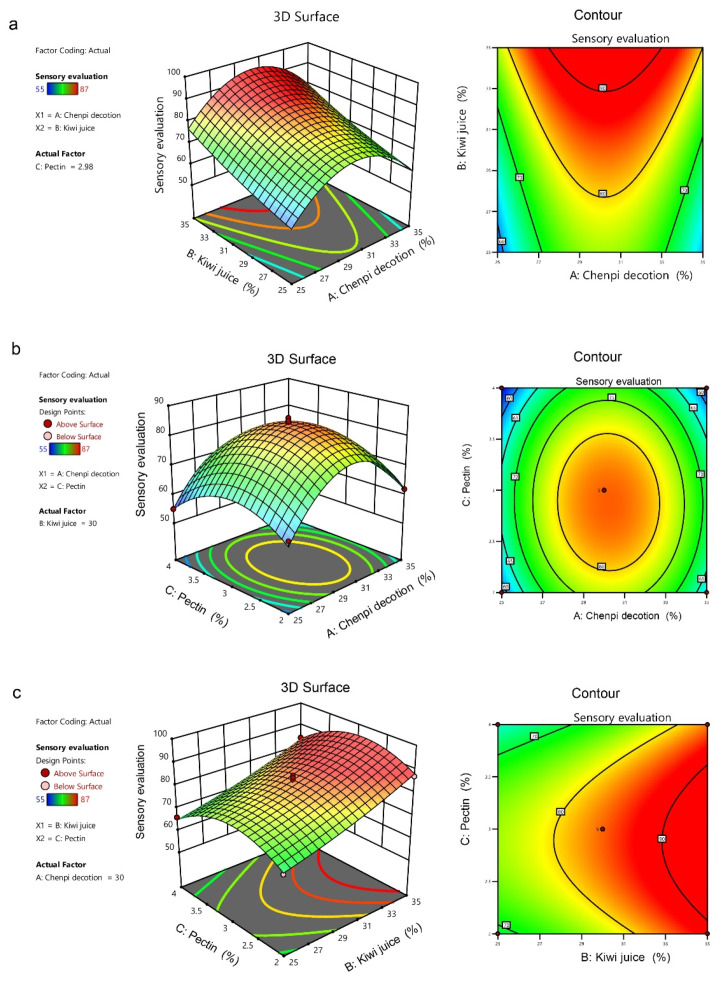
Response 3D surfaces and contours of AB (**a**), AC (**b**), BC (**c**) interaction effects.

The 3D response surface and contour plots of the interaction effects of A-*chenpi* decoction (%), B-kiwifruit juice (%), and C-pectin (%) are shown in Figure 2. The steepness of the 3D response surface reflects the interaction between two factors, and the steeper the slope, the more significant the interaction. The shape of contour lines can reflect whether the interaction is significant or not; elliptical contour lines illustrated a significant interaction between two factors, and circular contour lines indicated little interaction. The 3D response surface of the sensory evaluation between A-*chenpi* decoction and C-pectin was steep, but the contours were close to circular, indicating that the interaction between these two factors was not significant (Figure 2b). Although the contours between A-*chenpi* decoction and B- kiwifruit juice were close to an ellipse, the 3D response surface was not significantly steep, indicating that the interaction between these two factors was not significant (Figure 2a). Similar results were observed between B-kiwifruit juice and C-pectin (Figure 2c). The results were consistent with the above ANOVA results.

Finally, the best formulation was generated based on the regression model, and the results were validated. The highest sensory evaluation score of 95.20 was predicted with 30.26% addition of *chenpi* decoction (A), 35% addition of kiwifruit juice (B), and 2.88% addition of pectin (C). The actual score for the product based on the best formulation was 94.2 after evaluation by the sensory evaluation team members, which was similar to the predicted value.

#### 3.1.3. 3D Printing of FKJ

**Figure 3:**
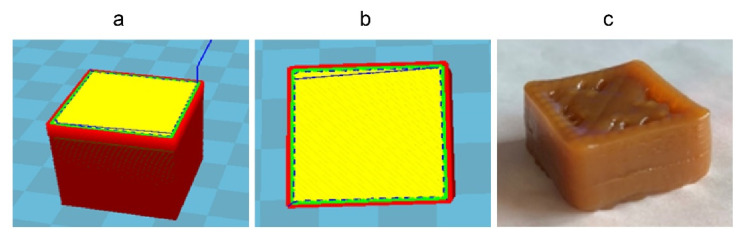
Three-dimensional printing of FKJ. (**a**) Scheme of the 3D printed cuboid, 2.5 × 2.5 × 1.25 cm^3^, designed in Cura15.02.1 software. (**b**) Transversal view of the 3D designed object. Cross-section of one layer showing the printhead pathway; the yellow part in the middle is the printing infill part, and the slash in the middle represents the infill patterns. (**c**) 3D printing of a cuboid with food-ink made of best formulation.

3D food printing experiments were designed to investigate whether FKJ could be used as a printing material for extrusion printing. After the adjustment and testing of printing parameters, the expected food shape was printed successfully according to the food shape designed by the software. Figure 3a shows the design of the print shape (cuboid) of FKJ in the Cura 15.02.1 software. The cross-section of the print shape design is also shown (Figure 3b) and provides a visualization of the filling pattern during printing (rectilinear filling with a diagonal orientation). Finally, the printing of FKJ was completed (Figure 3c). Although the middle of the shape is slightly sunken, the printed FKJ sample had a high degree of matching with the designed shape.

### 3.2. Determination of Nutrients and Bioactive Components of FKJ

#### 3.2.1. Nutritional Evaluation

The main nutritional composition of FKJ made with the optimal formula was deter-mined (Table 5). The results showed that FKJ was almost fat-free (0.1%), contained a small amount of protein (0.61%), and had a carbohydrate content of 20.5%, with a total energy of 369 kJ (88.28 cal) in 100 g of FKJ.

#### 3.2.2. Determination of Total Flavonoids (TF) and Phenols (TP) of FKJ

**Figure 4:**
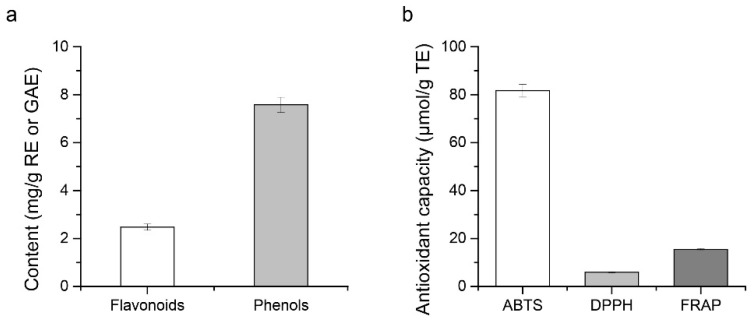
Bioactive substances (**a**) of FKJ and its antioxidant ability (**b**).

In order to analyze the content of biologically active substances in FKJ, TP and TF contents were tested as shown in Figure 4a. The results showed that the TP and TF contents were 7.58 ± 0.32 mg GAE/g and 2.47 ± 0.13 mg RE/g, respectively.

### 3.3. Determination of Antioxidant Capacity of FKJ In Vitro

As shown in Figure 4b, FKJ possessed 81.65 ± 2.65 μmol/g TE ABTS and 5.94 ± 0.21 μmol/g TE DPPH radical scavenging capacity, and 15.49 ± 0.21 μmol/g TE FRAP reducing capacity.

### 3.4. Bioactive Effects of FKJ on Healthy Mice

#### 3.4.1. FKJ Alleviated Body Weight Gain and Fat Accumulation in Mice

**Figure 5:**
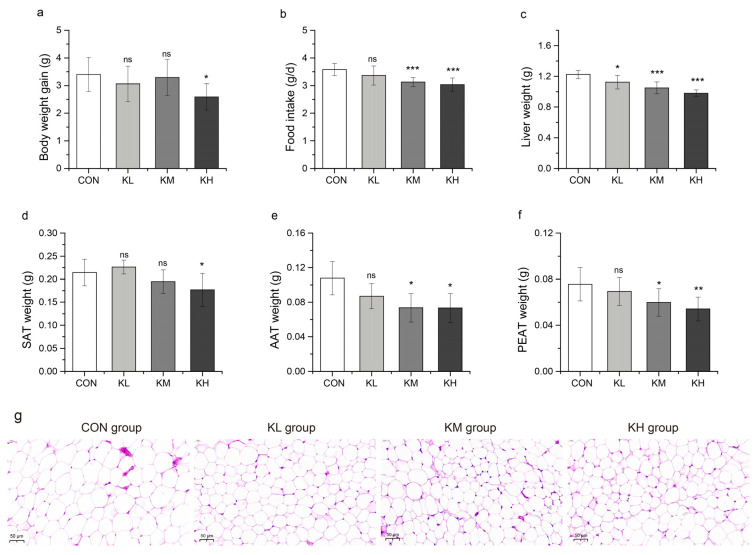
FKJ alleviated body weight gain and fat accumulation in mice (n = 7–10). (**a**) Body weight gain (in grams) after 8 weeks of FKJ supplementation (the control group was expressed as CON, and the groups supplemented with low-dose, medium-dose, and high-dose FKJ were expressed as KL, KM, and KH, respectively). (**b**) The average daily food intake of mice during the whole experimental period. (**c**) Liver weight, (**d**) subcutaneous adipose tissues (SAT) weight, (**e**) abdominal adipose tissues (AAT) weight, (**f**) perirenal adipose tissues (PEAT) weight. (**g**) The observation of AAT by H&E staining of four treatment groups (40×). * *p* < 0.05; ** *p* < 0.01; *** *p* < 0.001; and ns *p* > 0.05. Compared with CON group.

As shown in Figure 5, body weight was significantly reduced in the KH group compared to the CON group (*p* < 0.05). Interestingly, mice showed a dose-dependent decrease in food intake, liver weight, SAT weight, AAT weight, and PEAT weight after administration of FKJ compared to the CON group (Figure 5a–f). The liver weights in all three FKJ treatment groups were significantly lower than those in the CON group (*p* < 0.05, *p* < 0.001, and *p* < 0.001, respectively). The food intake of mice in the KM and KH groups was significantly lower than that of mice in the CON group (*p* < 0.001 and *p* < 0.001), while there was no remarkable effect in the KL group (Figure 5b). Compared with the CON group, the weights of SAT, AAT, and PEAT showed a significant decline in the KH group (*p* < 0.05, *p* < 0.05, and *p* < 0.01, respectively). The weights of AAT and PEAT in the KM group were significantly lower than those in the CON group (*p* < 0.05 and *p* < 0.05), while the weight change in the KL group was not significant (Figure 5e, f). Consistently, the histopathological examination of AAT also revealed a dose-dependent inhibition of adipocyte enlargement after FKJ supplementation in mice, as shown in Figure 5g. These results suggested that FKJ could slow down weight gain and fat accumulation in mice, which might be related to the control of appetite in mice by FKJ supplement.

#### 3.4.2. FKJ Reduced the Risk of Hyperlipidemia and Inflammation

**Figure 6:**
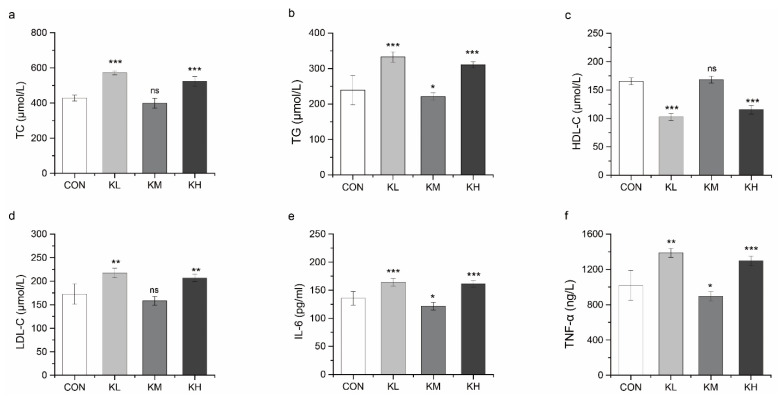
FKJ reduced the risk of hyperlipidemia and inflammation. Concentrations of total cholesterol (TC) (**a**), triglycerides (TG) (**b**), high-density lipoprotein cholesterol (HDL-C) (**c**), low-density lipoprotein cholesterol (LDL-C) (**d**), interleukin-6 (IL-6) (**e**), and tumor necrosis factor-alpha (TNF-α) (**f**) in serum (n = 7–10). The control group was expressed as CON, and the groups supplemented with low-dose, medium-dose, and high-dose FKJ were expressed as KL, KM, and KH, respectively. * *p* < 0.05; ** *p* < 0.01; *** *p* < 0.001; and ns *p* > 0.05. Compared with CON group.

Abnormally elevated serum levels of TC, TG, and LDL-C, as well as reduced serum levels of HDL-C caused by disorders of lipid metabolism are always associated with a high prevalence of hyperlipidemia [40,41]. A noticeable phenomenon was observed in the results for the serum biochemical indexes (Figure 6a–f). Among the three different doses of FKJ, the medium dose (KM group) showed a remarkable effect in regulating lipid metabolism and was significantly different from the other two groups. Compared with the CON group, the level of serum TG in the KM group was significantly decreased (*p* < 0.05), while changes in TC, HDL-C, and LDL-C were not significant (Figure 6a–d).

IL-6 and TNF-α are pro-inflammatory factors that cause inflammatory responses in the body [42]. The concentrations of serum IL-6 and TNF-α in the KM group were significantly lower than those in the CON group (*p* < 0.05) (Figure 6e,f). In contrast, the KL and KH groups showed the opposite results in comparison with the KM group.

#### 3.4.3. FKJ Enhanced Antioxidant Capacity In Vivo

**Figure 7:**
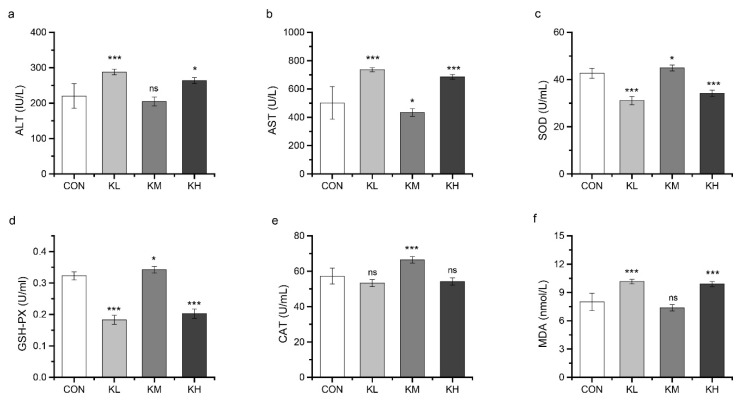
FKJ enhanced antioxidant capacity in vivo. The serum levels of aminotransferase (ALT) (**a**), aspartate transaminase (AST) (**b**), superoxide dismutase (SOD) (**c**), glutathione peroxidase (GSH-PX) (**d**), catalase (CAT) (**e**), and malonaldehyde (MDA) (**f**). The control group was expressed as CON, and FKJ supplemented with low-dose, medium-dose, and high-dose were expressed as KL, KM, and KH. * *p* < 0.05; *** *p* < 0.001; and ns *p* > 0.05. Compared with CON group.

ALT and AST are representative indicators of liver function that reflect the health conditions of the liver. Compared with the CON group, the serum AST levels in the KM group were significantly decreased (*p* < 0.05), but the serum levels of ALT (Figure 7a,b) were not affected by the medium FKJ dose. In contrast, the KL and KH groups showed a significant increase compared to the CON group, in both ALT and AST. These results suggested that FKJ supplement at the medium dose had a significant preventive effect against liver injury.

The MAD level, SOD activity, GSH-PX activity, and CAT activity in serum are related with the body’s antioxidant capacity. As shown in Figure 7, SOD, GSH-PX, and CAT were significantly increased in the KM group compared to their levels in the CON group (*p* < 0.05), whereas MDA levels were not significantly different between the two groups (Figure 7c–f). In contrast, MDA was significantly increased and the SOD, GSH-PX, and CAT were significantly decreased in the KL and KH groups. These results indicated that the antioxidant capacity of mice might be effectively improved by supplementation with FKJ at medium dose.

## 4. Discussion

In this study, a kind of functional kiwifruit jelly snack with low calories and antioxidant activity was developed using *chenpi*, kiwifruit juice, and citrus pectin. Single-factor experiments were used to explore the effects of various components and determine the range of addition of each ingredient. Then, a response surface experiment was designed to obtain an optimal food production formulation, which was combined with 3D food printing technology to obtain jelly products of specific shapes rapidly.

*Chenpi* is the main functional ingredient, which might contribute to an FKJ product with better health benefits. In the single-factor experiment, we observed that with increases in the concentration of *chenpi*, the sensory evaluation score of FKJ was the highest at a concentration of 30%, and then decreased significantly. This may be because the bitter substances (limonoids) contained in *chenpi* increased [43]. Kiwifruit juice is the source of most vitamin C and phenolic substances, which neutralize the bitterness of *chenpi*. LMP can form a gel in the presence of calcium ions [44], and the effect of pectin concentration on FKJ was determined in this study. Acosta, O. et al. have examined the effect of calcium concentration on jelly [45], which was not discussed in this study because changes in calcium concentration did not have a significant impact on our products (in the preliminary experimental results, not shown in this study).

Currently, jelly products are produced using traditional plastic molds or silicon molds [46]. However, these molds are often used to produce large quantities of simple product shapes, and might also cause environmental pollution problems [47]. Compared to molds, 3D food printing technology offers the advantage of customizing jelly products according to consumers’ needs for shape, color, flavor and even texture, which is difficult to achieve in the production of molds [48]. Three-dimensional food printing is an achievement of the application of 3D printing technology in the food manufacturing industry [49] that allows for personalized food customization based on consumer gender, age, physical/health status and nutritional needs, while also maximizing time and material savings [50,51]. Extrusion printing is generally the most common mode today in this industry [52], which has been successfully printing paste-like foods such as mashed potatoes for dysphagic patients [53] and fruit-based snacks that can meet the nutritional needs of children [54]. In our study, the optimized FKJ met the requirements of extrusion printing and enabled successful printing of the expected food shape. Although there was a slight deviation between the printed sample and the target shape (a slight depression in the middle), this may have been due to the deposition deformation of the printed material [55]. At present, we are still in the early stage of exploratory experiments and have only designed a simple jelly shape for 3D printing; more complex and diversified jelly shapes will be obtained in future studies. At the same time, with the increasing popularity of 3D printing technology in the food industry, it will be easier and faster to obtain customized food in the future. Personalized customization will be a development trend in the future. While the production of ordinary molds to design jelly shapes is more affordable at this stage, 3D food printing is more advantageous for customized foods, as even trained masters require more time and effort to satisfy complex customization requirements.

The Code of Federal Regulations of The U.S. (Title 21, part 101, chapter 1, subchapter B, 2022) states that the term “low-calorie” is used provided that the reference amount of the food is normally 30 g or less, or 2 tbsp or less, and does not exceed 40 calories in each reference amount [56]. In the low-calorie jelly studies, Khouryieh, H. A. et al. developed a low-calorie grape jelly offering 10 calories in one serving (1 tbsp or 15 g) [28]; Acosta, O. et al. developed a kind of blackberry jelly that provided less than 8 calories per serving [57] and a mixed fruit jelly that provided less than 12 calories per serving (1 tbsp or 15 mL) [45]. In our study, based on the comprehensive consideration of various components of FKJ, the recommended daily intake was less than 30 g given that 100 g of FKJ provided 88.28 calories, and each serving (less than 30 g) provided less than 26 cal. Therefore, FKJ could be considered as a low-calorie food.

Tylewicz, U. et al. developed two kinds of healthy snacks (fruit leathers and bars) based on kiwifruit with a TP content of about 922–1346 mg GAE/100 g dry weight (DW) and a TF content of about 0.45–4.92 ± 0.33 mg quercetin/100 g DW [18]. Similarly, Donno, D. et al. developed a freeze-dried fruit product from kiwifruit containing TP of 210.9 mg GAE/100g DW [17]. Our results showed that FKJs were rich in phenolic compounds (7.58 ± 0.32 mg GAE/g) and flavonoids (2.47 ± 0.13 mg RE/g). Both *chenpi* decoction [11] and kiwifruit juice [58,59] are rich in phenolic compounds, and flavonoids are the most abundant phenols [60]. Ercan Bursal et al. [61] produced a lyophilized aqueous extract of kiwifruit and determined a TP content of 16.67 ± 2.83 mg GAE/g. In comparison, the TP content of FKJ was 7.58 ± 0.32 mg GAE/g, probably due to the use of kiwifruit juice in our preparation, which may have lower TP content than the aqueous extract of kiwifruit. Jin Wang et al. [62] determined that the TP content of kiwifruit juice was 0.58 ± 0.01 mg GAE/mL, which was much lower than that of fresh kiwifruit. The TF content of fresh kiwifruit ranged from 0.82–1.61 mg RE/g fresh weight [58], while 2.47 ± 0.13 mg RE/g was detected in FKJ. The possible reason might be that another material in FKJ, *chenpi* decoction, also contains large amounts of flavonoids [63].

ABTS, DPPH, and FRAP assays are widely used methods for the determination of antioxidant activity in vitro [64]. The ABTS assay is based on the production of blue/green ABTS^+^, which is suitable for hydrophilic and lipophilic antioxidant systems; the DPPH analysis uses free radicals dissolved in organic media and is, therefore, suitable for hydrophobic systems [65]; the FRAP method depends on the ability of the antioxidant to reduce Fe^3+^ to Fe^2+^, which is bound to the ligand, producing an intense navy blue color [66]. Due to the different principles of the three determination methods, it is necessary to use them together to obtain more accurate results for antioxidant capacity. Our results showed that FKJ possessed 81.65 ± 2.65 μmol/g TE ABTS and 5.94 ± 0.21 μmol/g TE DPPH radical scavenging capacity, and 15.49 ± 0.21 μmol/g TE FRAP reducing capacity. The obvious differences in the results of the three methods may be related to the different reactions of various antioxidants in FKJ with the radicals or iron ions used [64]. In addition, since the ABTS assay better reflected the highly pigmented and hydrophilic antioxidants than the DPPH assay [67], the scavenging of ABTS radicals by FKJ was significantly higher than that of DPPH radicals. A similar phenomenon was also observed in the experiments of Thaipong, K. et al. [37] and Anna Floegel et al. [67]. The antioxidant capacity of fruit or vegetable extracts measured by ABTS was significantly higher than that measured by DPPH and FRAP in their results. Antioxidant activity is positively correlated with phenolic content [8] and vitamin C, which are abundant in kiwifruit juice [13] and *chenpi* [68]. Kiwifruit is generally considered to be a fruit with high antioxidant activity [69]. The antioxidant capacity of different kiwifruit varieties varies considerably, and studies by Y.-S. Park et al. showed that the ABTS radical scavenging capacity of kiwifruit ranged from 22.9–109.0 μmol TE/g DW, 8.49–102 μmol TE/g DW in the DPPH assay, and 11.0–94.4 μmol TE/g DW in the FRAP assay [70]. Most of our results fall in this range, and these results were expressed as fresh weight of FKJ. Thus, it can be suggested that FKJ has certain antioxidant activity in vitro.

We observed a slowdown in weight gain and a decrease in fat accumulation in a dose-dependent manner in healthy mice after long-term intake of FKJ. One of the main effects may have been influenced by *chenpi* [71]. Guo, J et al. illustrated that *chenpi* extract can reduce adipogenesis effectively during the differentiation of 3T3-L1 adipocytes [72]. Moreover, a study showed that *chenpi* extract (rich in PMF) could effectively attenuate obesity and hepatic steatosis caused by a high-fat diet [73]. In addition, kiwifruit juice, one of the main ingredients in FKJ, may play an essential role because of its abundance of vitamin C and flavonoids [74,75]. A study carried out by Alim, A. et al. showed that intervention with two kiwis per day was effective in reducing body weight and liver weight in healthy rats [76]. Furthermore, pectin is a soluble dietary fiber that improves metabolic homeostasis while enhancing satiety [77,78], which may explain the decrease in food intake during FKJ supplementation [79]. In short, the anti-obesity effects and health benefits exerted by FKJ in healthy mice may be the result of the combined effect of several main components.

A growing number of studies have shown that kiwifruit and its related processed products are able to improve lipid metabolism and enhance antioxidant capacity [80]. The study of Alim, A. et al. suggested that supplementation with kiwifruit significantly reduced TC and TG and increased HDL-C levels in rats; meanwhile, the lipid peroxidation was improved by decreasing MDA concentration and increasing GSH-PX and SOD activity [76]. Similarly, the results of a clinical study also showed that consuming kiwifruit juice increased the activity of antioxidant-related enzymes in the serum of patients and reduced markers of inflammation [81]. Further, kiwifruit juice fermented with probiotics also showed hypolipidemic and anti-oxidative effects in mice on a high-fat diet by improving the corresponding indexes in the serum [82]. In our study, similar effects were also observed in mice administered with a medium dose of FKJ, and *chenpi* may also play an important role. *Chenpi* is rich in polymethoxyflavones (PMFs) [83], which could exert anti-inflammatory and antioxidant effects [8]. In our study, low doses of FKJ did not show similar health effects because they fell below the threshold of usefulness. Notably, the same effect was not obtained in mice ingesting high doses of FKJ, which was supposed to be a counter effect of high doses. Thus, supplementation with moderate doses of FKJ (medium dose in this study) can effectively improve the lipid profile and anti-inflammatory and antioxidant abilities.

## 5. Conclusions

In conclusion, functional kiwifruit jelly with *chenpi* was developed through single-factor and response surface experiments. FKJ was rich in phenols and flavonoids, while being low in calories, and possessed good antioxidant capacity. FKJ can also be personalized using 3D food printing technology. In addition, in vivo experimental results illustrated that daily supplementation with FKJ at an appropriate dose (medium dose) could moderately reduce weight gain and fat accumulation, improving serum lipid profiles, relieving inflammation and liver damage, and improving antioxidant capacity. However, it should be noted that FKJ intake is not recommended above the high dose in this study, which may cause adverse effects. FKJ could meet the needs of modern people for nutrition and health and also promote the processing and utilization of natural products, and has good development prospects in the functional food industry.

## Figures and Tables

**Table 1:** Indicators of sensory evaluation of FKJ.

Indicators	Scoring Criteria
Color (10 points)	Dark brown with more uniform color (7–10 points). Brownish with uniform color (4–6 points). Light brown with uneven color (0–3 points).
Tissue state (30 points)	Excellent toughness, uniform texture without bubbles, fine and smooth (21–30 points). Good toughness, the texture is basically uniform and fine, and a small amount of bubbles are allowed (11–20 points). Less tough, uneven texture, with many bubbles (0–10 points).
Flavor (30 points)	Has a strong and fresh *chenpi* aroma and kiwifruit flavor with no bad odor (21–30 points). Has a light and clear *chenpi* aroma and kiwifruit flavor (11–20 points). Does not have a clear aroma of *chenpi* or kiwifruit flavor, and has an undesirable odor (0–10 points).
Taste (30 points)	Has a smooth and delicate taste, with the best sweet and sour taste and no obvious bitterness (21–30 points). Has a basic smooth taste, with basic sweet and sour taste, and the presence of post-bitterness or slight acidity (11–20 points). Tastes rough, too sour or too sweet, with a heavy bitterness (0–10 points).

**Table 2:** Variables of single-factor experiments and response surface methodology.

Factors of SFE	Variables					
Additions of *chenpi* decoction (%)	10	15	20	25	30	35
Additions of kiwifruit juice (%)	15	20	25	30	35	40
Additions of pectin (%)	1	2	3	4	5	6
**Independent Variables of RSM**	**Levels**					
	**−1**		**0**		**1**	
A: additions of *chenpi* decoction (%)	25		30		35	
B: additions of kiwifruit juice (%)	25		30		35	
C: additions of pectin (%)	2		3		4	

**Table 3:** Box–Behnken response surface factors and levels.

Run	A: *Chenpi* Decoction (%)	B: Kiwifruit Juice (%)	C: Pectin (%)	R: Sensory Evaluation
1	25	25	3	57
2	35	25	3	61
3	30	30	3	85
4	30	30	3	86
5	35	30	2	62
6	25	35	3	76
7	25	30	4	55
8	35	35	3	81
9	30	35	2	87
10	30	25	4	66
11	30	25	2	68
12	30	30	3	84
13	30	30	3	82
14	35	30	4	55
15	30	35	4	84
16	25	30	2	60
17	30	30	3	83
18 ^1^	30.26	35	2.88	95.20

^1^ The best formulation and the predicted values of sensory evaluation based on the model.

**Table 4:** Variance analysis of fitted model ^1^.

Source	Sum of Squares	df	Mean Square	F-Value	*p*-Value	
Model	2389.49	9	265.5	81.69	<0.0001	significant
A-*Chenpi* decoction	15.13	1	15.13	4.65	0.0679	
B-Kiwifruit juice	722	1	722	222.15	<0.0001	
C-Pectin	36.13	1	36.13	11.12	0.0125	
AB	0.25	1	0.25	0.0769	0.7895	
AC	1	1	1	0.3077	0.5964	
BC	0.25	1	0.25	0.0769	0.7895	
A²	1181.32	1	1181.32	363.48	<0.0001	
B²	9.47	1	9.47	2.91	0.1315	
C²	360.26	1	360.26	110.85	<0.0001	
Residual	22.75	7	3.25			
Lack of Fit	12.75	3	4.25	1.7	0.3038	not significant

^1^ R² = 0.99, Adj. R² = 0.98, C.V. (%) = 2.49, adequate precision = 23.96.

**Table 5:** Nutritional composition of the optimal FKJ ^1^.

Project	Per 100 g	NRV% ^2^
energy	369 (kJ) ^3^	4%
protein	0.61 (g)	1%
fat	0.1 (g)	0%
carbohydrate	20.5 (g)b	7%
sodium	19 (mg)	1%
ash	0.65 (g)	-
moisture	80.4 (g)	-

^1^ Data reported as means ± standard deviation (n = 3). ^2^ NRV% value calculation method and format reference GB 28050-2011. ^3^ Determined by calculation.

## Data Availability

Data are contained within the article.
